# A comprehensive scoping review of existing carotid duplex ultrasound scanning and reporting protocols: identifying gaps and opportunities for standardization of practice in low-income countries

**DOI:** 10.1007/s40477-025-01064-1

**Published:** 2025-08-28

**Authors:** Theonille Mukabagorora, Linda Mbonambi, Zarina Lockhat, Amiable Musafiri, Ramadimetja Mable Kekana

**Affiliations:** 1 Radiography Department, School of Health Sciences, University of Pretoria, Pretoria, South Africa; 2 Faculty of Health Sciences Library, University of Pretoria, Pretoria, South Africa; 3 Radiology Department, Faculty of Health Sceinces, University of Pretoria, Pretoria, South Africa; 4 School of Medicine and Pharmacy, University of Rwanda-College of Medicine and Health Sciences, Kigali, Rwanda; 5 Department of Medical Imaging, School of Health Sciences, University of Rwanda-College of Medicine and Health Sciences, Kigali, Rwanda

**Keywords:** Carotid vessels/Extracranial vessels, Scanning/Reporting guidelines, Protocols/Criteria, Duplex ultrasound, Atherosclerosis/Plaques/Stenosis

## Abstract

**Background:**

Duplex carotid ultrasound is a non-invasive imaging test that is essential for assessing carotid artery disease, particularly in determining the presence and severity of atherosclerosis and the risk of cerebrovascular events. However, the interpretation of ultrasound results can differ widely due to variations in diagnostic criteria, lack of standardization in scanning methods, and differing approaches to evaluating carotid intimal medial thickness (CIMT), plaques, and stenosis. These inconsistencies create challenges in clinical practice and the reproducibility of results. While various protocols for CIMT, plaque measurement, and stenosis grading are available in the literature, a standardized global protocol is still lacking. This article presents a scoping review of current carotid duplex ultrasound scanning and reporting protocols. It aims to identify existing protocols and guidelines for carotid ultrasound scanning and reporting, highlight variations, and propose a standardized approach to enhance diagnostic accuracy and clinical outcomes in carotid atherosclerosis.

**Methods:**

Using predefined search protocols developed in collaboration with the specialized librarian from the University of Pretoria, we searched different databases and gray literature to identify existing protocols on carotid ultrasound including scanning technique, carotid intimal medial thickness, carotid plaques, and carotid stenosis grading. Due to limitations in the length of the review, and the existence of a large number of protocols, this review focused on the protocols developed and published by institutions and professional societies rather than individual articles.

**Results:**

Among 2496 articles identified from databases and 22 from other sources, 17 articles met the criteria for this review. The most common criteria that was found following this review are those established by the Society of Radiologists in Ultrasound (SRU) in 2003. The researchers further noted that these criteria were, however, been criticized for relying heavily on peak systolic velocities, a measurement that, when used alone, can be misleading. Literature further demonstrated that other researchers and professional societies have called for consensus on unified criteria for the diagnosis of carotid atherosclerosis.

**Conclusion:**

While carotid ultrasound is an important, non-invasive, and widely available method for evaluating carotid atherosclerosis, the variations in scanning techniques, measurements, and cut-off values underscore the need for standardized diagnostic criteria. Standardization is essential to provide consistent patient care and ensure accurate examination in various clinical settings. This will lead to the reduction of stroke and cardiovascular incidence which are the leading causes of death worldwide and more prevalent in low-income countries.

## Introduction

Ischemic stroke is attributable to about 85% of all strokes [1]. Atherosclerotic vascular disease includes a thickening of the innermost arterial layer (intima), as well as a more complicated focal protrusion into the arterial lumen (atherosclerotic plaque) [2]. Duplex carotid ultrasound is used in clinical practice as the first test to detect carotid artery atherosclerosis [3]. The path to understanding carotid disease has evolved over time, and medical imaging plays an important role in diagnosis, risk stratification, and treatment planning [4]. In the early 1980s, carotid artery studies relied on invasive angiography, either with direct carotid puncture or femoral catheterization [4]. The history of carotid stenosis started long before the modern medicine, in 1658, Wepfer came up with the first suggestion of a link between symptoms of cerebral arterial insufficiency and carotid disease [5]. It took centuries of medical evolution, with contributions from pioneers like Virchow and others, to explain that carotid atherosclerosis was a crucial factor in cerebrovascular accidents [4].

In the twentieth century two main clinical trials that initiated the grading criteria for carotid stenosis were conducted, one is the North American Symptomatic Carotid Endarterectomy Trial (NASCET), which was conducted between 1987 and 1996, and the European Carotid Surgery Trial (ECST) which was conducted from 1981 to 1994. The two big clinical trials were somehow parallel but in different locations and used different methodologies in the calculation and estimation of carotid stenosis grading. To determine the degree of stenosis The NASCET method compares the stenosed lumen to the normal lumen of the distal internal carotid artery (ICA), whereas the ECST determines the degree of stenosis in relation to the original lumen [6]. The use of two separate angiographic methodologies at the beginning, the North American Symptomatic Carotid Endarterectomy Study and the European Carotid Surgery Trial was most likely the primary source of initial uncertainty in developing valid and accurate universal duplex ultrasonography criteria [6].

In the mid-1970s, a team of doctors and bioengineers at the University of Washington, led by Dr. D Eugene Strandness, Jr., developed a prototype duplex scanner that integrated B-mode imaging and pulsed Doppler flow detection in a single instrument. This work resulted in the University of Washington duplex criteria for carotid stenosis published in 1987 [7, 8]. The initial University of Washington criteria were widely adopted by vascular laboratories and clinicians for internal carotid artery (ICA) stenosis assessment, as well as a starting point for refining interpretation criteria based on internally validated criteria [9].

Since the original University of Washington carotid duplex criteria were published in the 1980s, there have been ongoing efforts by researchers and professional societies to harmonize carotid diagnostic criteria, which continues till today. Unfortunately, this refinement has resulted in a proliferation of various diagnostic criteria and less standardization of criteria interpretation across vascular testing facilities, even facilities in the same country or the same region [10].

In 2003 the international society of Radiologist released a consensus on carotid stenosis grading with Duplex ultrasound following the results of several clinical trials. These criteria until now are the most common criteria used in many laboratories. However, several criticisms have been raised by researchers claiming that these criteria rely only on the velocities of blood to estimate the stenosis but do not consider the hemodynamic changes and the plaque characterization [11, 12]. Individual vascular laboratories in some developed countries have so far initiated internal validation of scanning protocols and interpretation criteria leading to varying sensitivity and specificity of the carotid stenosis grading. Kelly R. Byrnes suggested the adoption of the consensus in the absence of internal validation since it is the most commonly adopted method [13].

The absence of internationally accepted carotid ultrasonography standards criteria for determining the degree of stenosis has led to inconsistency and variation in practice across the globe. In 2010 the multi-parametric Deutsche Gesellschaft fu¨r Ultraschall in der Medizin ultrasound criteria (DEGUM) were proposed by German experts, they tried to bring in some additional criteria such as hemodynamic parameters and advocates for a multiparametric approach that considers both morphological and hemodynamic factors to overcome limitations associated with using Peak Systolic Velocity (PSV) in isolation [6]. Despite all of these efforts, a wide range of practice patterns remain, with inconsistencies in the standardization of practice. The DEGUM criteria of 2010 was a revision of the initial criteria which was developed in 1986. One year later the Strandness criteria came into formal use in 1987 although majority of studies report that the Strandness Criteria were published first, it came into use after the initial formulation of the DEGUM Criteria [8, 14].

In 2010, the Intersocietal Accreditation Commission (IAC) for vascular testing conducted research to evaluate the variance in carotid criteria which were being used to grade the carotid stenosis. They postulated that instead of improvement in standardization, there were more diagnostic criteria in use even in the accredited facilities and less agreement as to which criteria should be preferred over the others for carotid stenosis grading and interpretation. However, the SRU criteria, were the most common criteria used by vascular facilities [10]**. **Two years later in 2012, the IAC conducted a survey on medical and technical staff of accredited vascular laboratories to get feedback on whether the IAC should provide recommendations as to which criteria should be used for carotid artery stenosis interpretation. In this survey, 68% of participants felt that there should be only one set of diagnostic criteria for ICA stenosis [9].

The variation in carotid ultrasound diagnostic thresholds, were also examined in USA, testing protocols from 338 accredited vascular testing centers. The internal carotid artery peak systolic velocity was used by all centers to assess carotid diseases, and revealed that 60 distinct PSV thresholds were in use. The PSV threshold for moderate (≥ 50%) stenosis varied from 110 to 245 cm/s (median, 125 cm/s), and the threshold for severe (≥ 70%) stenosis varied from 175 to 340 cm/s (median, 230 cm/s) [15]. The variation in carotid stenosis interpretation facilities undermines the relevance of this essential diagnostic tool. The diagnosis that individuals receive can change based on which vascular ultrasound center performed the carotid ultrasound even in accredited vascular centers [15]. Majority of protocols focus on velocity criteria and put less focus on carotid IMT and plaques characterization. The American society of echocardiography is one of the society that provided details on the scanning techniques and plaque characterization. [16, 17].

The confusion related to carotid ultrasound scanning and reporting is worse in low resource settings where the number of experts is even limited and the possibility of having internal validation is less. Patients with the same degree of carotid stenosis may have different results and different recommendations for disease management from different facilities. Standardizing carotid duplex ultrasound diagnostic criteria will address these concerns while also improving the accuracy, validity, adaptability, and utility of duplex sonography for carotid disease diagnosis. It is important to note that performing carotid duplex ultrasonography requires consistent use of validated criteria.

The researchers of the current article conducted a systematic literature search to identify the existing validated scanning and reporting protocols for carotid ultrasound atherosclerosis. The aim was to further identify those that emphasized on all aspects of Duplex carotid ultrasound including *technical considerations, intimal medial thickness, carotid plaques and carotid stenosis*. This scoping review did not go deep into each of the mentioned elements however, it looked at whether the articles touched on these elements either in details or in brief.

## Methods

### Search strategy

*To identify the available guidelines and protocols for carotid ultrasound, extensive literature search was done mainly in four databases.* We followed Preferred Reporting Items for Systematic Reviews and Meta-analysis Extension for Scoping Reviews (PRISMA-ScR) for reporting the results**.** [18] *Selection of the guidelines was done by the principal investigator in collaboration with the research team. The systematic literature search was done using the following search strategy: ( "Report guide" OR "scanning guide" OR "screening tool" OR "protocol" OR "guidelines") AND ( "carotid vessels" OR "extracranial" OR "internal carotid") AND ( "ultrasound" OR "scanning" OR "sonography") AND ( "stenosis" OR "atherosclerosis" OR "plaques" OR "ischemic stroke"), four search engines were mainly used; PubMed, Scopus, web of science and ProQuest central.* To avoid missing any important protocol addition individual search was done on Hinari, Google Scholar and other grey literature. The search process was done based on the predefined search strategy in collaboration with an *experienced librarian from the University of Pretoria* from January 2024 to August 2024.

#### Selection criteria

We included studies that developed scanning or reporting guides or protocols for carotid ultrasound for either stenosis grading or any degree of atherosclerosis. Due to the existence of multiple carotid stenosis criteria, we focused on the studies that developed scanning and/or reporting protocols, not those that were criticizing or just reviewing the existing protocols. The language was limited to English and French to ensure that we understood the protocols. The publication year was left open to get the very initial protocols that were published long ago and to understand the root cause of the existing non-standardization of carotid ultrasound protocols. To ensure the validity of the protocols we only considered the study that was developed at the national level, regional level, protocols that were developed by a university, a professional society, or joint initiative between professional societies. We excluded studies that developed protocols or based on the individual studies or researcher(s) not on behalf of an institution or any other professional body.

#### Data collection and analysis

Literature was systematically reviewed. From the selected studies, the following data were extracted: First Author name and affiliating institution or body, publication year, duplex ultrasound cut-off criteria reported (PSV ICA, IMT) whether plaque measurements or plaque morphology was considered, where the technical consideration or scanning technics were described.

## Results

**Fig. 1:**
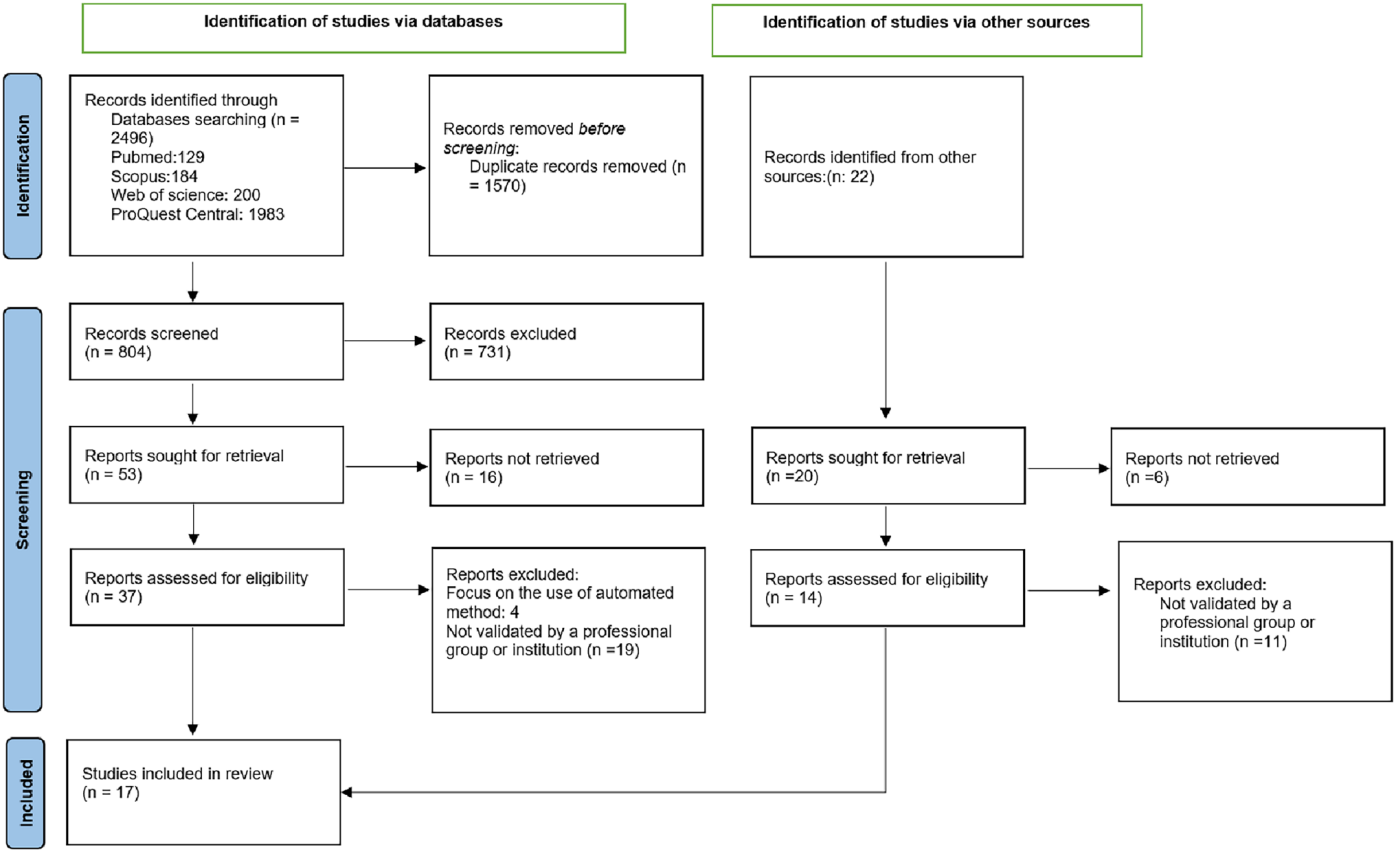
PRISMA flow diagram for studies selection

This scoping review has documented the highlights of existing protocols on carotid ultrasound including scanning and reporting protocols for carotid intimal medial thickness, carotid plaques, and carotid stenosis. Figure 1 summarizes the study selection and screening process, the database search retrieved 2496 records (PubMed, Web of Science, Scopus, and ProQuest Central). Through additional searches from Hinari and Gift and Google Scholar 22 additional records were found and assessed adding up to 2518 identified records in total. Among the records from the databases, we screened for Duplicate using the endnote software and Rayyan and remained with 804 records after removing the duplicates (1570). Titles and abstracts were screened using Rayyan, and 53 records were sought for retrieval and 16 of them were not retrieved. This led us to 37 records that were eligible for full-text screening, we assessed in full-text 37 based on the set criteria and 4 of them were focused on automated methods and 19 of them were excluded because they were not validated by any institution. We finally remained with 14 records from databases. On the other hand, of 22 records from other sources were screened manually, two of them were removed as duplicates,6 of them were not retrieved as full text, and 14 were assessed in full text, 11 of these were removed because they were not endorsed by any institution. Finally, 17 records including 14 records from databases and 3 records from other sources were eligible to be included in the study (Table 1). Figure 1 illustrates in detail the process we used for article selection.

**Table 1:** List of carotid ultrasound scanning and reporting protocols (Developed at the national, professional societies, institutional level)

	Authors	Institution/society or country if it is a national protocol	Year of publication	Cut off point for a normal ICA PSV	Lowest stenosis category	Cut off point for normal IMT	Comment about plaques morphology	Comment on post stenotic flow appearance	Details on the scanning Technics
1	D E Strandness Jr,D C Taylor [8]	University of Washington (Strandness Criteria	1987	125	1–15%	NA		Broadening	
2	Arning C et al. [14]	German Society ultrasound(DEGUM criteria)	1986, 2010	200	10%			Flow turbulence	
3	Edward G Grant [22]	Society of Radiologists in Ultrasound Consensus	2003	125	< 50%		Plaque presence and size		Details
4	Oates CP, et al. [23]	Joint recommendations for reporting carotid ultrasound investigations in the United Kingdom	2009	125	< 50%		Presence of plaque		
5	K. W. Beach et al [24]	Standardized ultrasound evaluation of carotid stenosis for clinical trials: University of Washington Ultrasound Reading Center	2010	125 with focus to EDV and angle					Details
6	Intersocietal Accreditation Commission (IAC) [10]	IAC Vascular Testing Communication: Updated Recommendations for Carotid Stenosis Interpretation Criteria,	2021	> 180 for 50%	> 50%				
7	M. Elwertowski et al. [19]	Standards of the Polish Ultrasound Society—update. Examination of extracranial carotid and vertebral arteries	2014	ICA,80–120, VA < 60	< 50	Details	Details	Details	Details
8	Jogestrand T [25, 26]	Standard criteria for grading carotid stenosis in Sweden	2002	< 110 for 46 degrees and < 130 for 60degrees	< 50				
9	J. A. Columbo et al. [27]	Dartmouth internal carotid artery Duplex ultrasound velocity Criteria	2018	< 220	50–59				Details
10	Marie Gerhard-Herman [28]	Guidelines for Noninvasive Vascular Laboratory Testing: A Report from the American Society of Echocardiography and the Society of Vascular Medicine and Biology	2013	< 125			size estimation		Details
11	UT Southwestern [21]	Ultrasound-carotid doppler complete evaluation by the UT Southwestern Department of Radiology	2022,2024	< 180	< 50		Size estimation	Details	Details
12	Amer M. Johri MD, et al.,, American society of echocardiography [17]	Recommendations for the Assessment of Carotid Arterial Plaque by Ultrasound for the Characterization of Atherosclerosis and Evaluation of Cardiovascular Risk: From the American Society of Echocardiography	2020	< 140	< 50	Details	Plaque size and grading		Details
13	American society of echocardiography [16]	A Consensus Statement from the American Society of Echocardiography Carotid Intima-Media Thickness Task Force Endorsed by the Society for Vascular Medicine	2008			Details			Details
14	Brazilian Society of Cardiology [29]	Recommendation Update for Vascular Ultrasound Evaluation of Carotid and Vertebral Artery Disease	2023	< 140 ICA, < 85 VA	< 59	Details	Details		Details
15	Society for vascular technology of Great Britain and Ireland [30]	Vascular Technology Professional Performance Guidelines Extracranial Cerebrovascular Duplex Ultrasound Examination	2019	< 125	< 50		Size		Details
16	Mannheim Carotid Intima-Media Thickness and Plaque Consensus [31]	An Update on Behalf of the Advisory Board of the 3rd and 4th Watching the Risk Symposium 13th and 15th European Stroke Conferences, Mannheim, Germany, 2004, and Brussels, Belgium, 2006	2013			Details	Details		Details
17	Luca Saba et al. [32]	Carotid Plaque-RADS: A Novel Stroke Risk Classification System	2024				Details		

*ICA* Internal carotid artery, *PSV* Pick systolic velocity, *EDV* End diastolic velocity, *IMT* Intimal medial thickness

### Characteristics of the studies

Herein, we present seventeen eligible studies (Table 1) that described carotid ultrasound scanning and/or reporting protocols at the national level, professional society level, or University level. The studies covered six different points, the authors, the institution or society that validated and endorsed the document, the year of publication, the cut-off point for the normal PSV level for ICA, the lowest category of stenosis reported, cut-off point for the IMT, plaque morphology, any detail about post stenotic hemodynamics, whether the study provided details about the scanning protocols. Most studies focused on the carotid stenosis grading, a few of them looked at plaque characterization and intimal medial thickness. Two studies reported on the PSV of the vertebral artery [19, 20].

The study by M. Elwertowski and co-authors presented the full list of the elements that were reported in this scoping review ranging from the Authors, Institution/society or country if it is a national protocol, Year of publication, the cut off point for a normal ICA PSV, lowest stenosis category, cut-off point for normal IMT, comment about plaques morphology, comment on post stenotic flow appearance, details on the scanning technics [19]. Three of seventeen discusses on almost all details except one element of post stenotic flow appearance [17, 20, 21]. The publication by the American society of echocardiography provided details about non-stenotic plaque grading based on the size of the plaque and the study graded them into 4 categories ranging from 0 to grade III [17]. In addition, the society provides detailed protocols for carotid ultrasound evaluation, focusing on carotid intima-media thickness (CIMT) measurement and plaque detection to assess cardiovascular risks (Table 2) [16].

**Table 2:** Recommended scanning protocol for evaluation of common carotid artery, carotid intima-media thickness, and detection of carotid plaques by the American Society of Echocardiography. [16]

Use/Purpose	View	Area of interest	Technique
Overview of vessel orientation, wall thickness, plaques, and surrounding structures	Transverse B-modes can of each segment	From proximal CCA through middle of the internal carotid artery	Notch of the transducer oriented toward the right of patient. Slowly advance the probe, keep the vessel in the center of the screen, show double lines on near and far walls
Verifies anatomic orientation and may identify significant stenosis if present	Internal and external carotid artery Doppler recordings	Pulsed wave Doppler of proximal 1 cm of each branch	Sample volume parallel to flow by beam steering and angle correction of ≤ 60 degrees
			If narrowing is observed, take pre- and post-stenotic velocities to document severity
Identification and description of plaques	Longitudinal plaque screen scan at least 3 different angles in each segment	Near and far walls of CCA, bulb, and internal carotid artery segments	Rotate 90 degrees from transverse plane with notch of transducer oriented toward the head of patient
			Circumferential plaque screen scan from anterior, lateral, and posterior imaging planes
			Return to transverse plane to corroborate maximum plaque size in orthogonal plane
			Document location and angle that plaque has greatest thickness
Segments for CIMT measurement	CIMT imaging	Distal 1 cm of each CCA	Longitudinal images from 3 imaging planes: anterior, lateral, and posterior
			Display clear images of distal CCA perfectly horizontal with double lines on near and far walls, indicating true perpendicular scanning plane
			Optimize transducer depth (usually 4 cm) to avoid slice thickness artifacts
			By convention, the right carotid artery is imaged first

*CCA* Common carotid artery; *CIMT* carotid intima-media thickness

### Discussion

The scoping review aimed to map the existing literature on the carotid ultrasound scanning and reporting protocols and guidelines. A total number of seventeen studies that met the criteria for inclusion in this study, revealed a significant variability in carotid ultrasound practices across the globe. The evidence suggests that the application of ultrasonography diagnostic criteria for carotid artery stenosis varies greatly. Until now, there is no universally accepted standard for grading carotid stenosis. Discrepancies in ultrasound criteria may result in clinically important misclassification of disease severity, resulting in improper or non-referral to revascularization centers, with the potential for negative serious consequences [33]. In addition the reporting format or template has not yet been unformalized, leading to discrepancy in elements of the carotid ultrasound reports, some are based on the velocities, others are based on the plaques characterization [34], and others combine different characteristics in what is known as multiparametric approach [23]. In this review we summarized the findings resulting from extensive literature review on existing carotid scanning and reporting protocols.

Columbo and his colleagues conducted a study to explore variation in the ultrasound diagnostic thresholds used to determine disease severity in the USA, and revealed that ICA PSV was used in all centers and PSV for moderate stenosis between 50 and 69% varied between 110 and 240 cm/sec with median of 125 cm/sec. [15] In our review the normal PSV for less than 50% stenosis ranged from 80 to 200 cm/sec, PSV of 125 cm /sec remained the most commonly used cut off point for a normal or less than 50% stenosis of internal carotid artery. [19, 35]

### Patient position and operator position

Many articles describing the carotid ultrasound do not touch the item of operator position. Majority of them focus on the patient position. There is no confusion about the patient position, the patient is positioned in supine and the head rotated toward the contralateral side. Lee explained the two commonly used positions of the operator in relation to the patient. He explains that there are two options for the operator position, the position where the operator is above the patients which allow the operator to use both hands to be free to move around the patient, and the second position being the usual position that is used for any other exam where the operator is on the lateral side of the patient. This position is known to be easier to manipulate the equipment buttons and allow the operator to use the right hand for both right and left carotid. In his conclusion he recommended the overhead position. The use of the pillow is controversial, some researchers say yes the pillow is important, others say that the pillow is not necessary as reported in the previous study [36]. None of the seventeen studies that fulfilled the inclusion criteria provided clarification on this aspect operator position.

### Equipment selection

The selection of proper angle of insonation has been extensively discussed in literature. Doppler angle of insonation has an important effect on spectral Doppler velocity measurements [37]. Some research indicates that the angle of insonation should consistently be set at 60 degrees, while other studies suggest that it can vary based on the patient, provided it remains below 60 degrees [38]. Moreover, some researchers reported the higher velocity when the angle of 60 degree used [39], Most studies indicate that using a fixed angle of insonation leads to consistent results, while even slight changes in the angle can cause significant discrepancies [33], One of the selected seventeen studies provided comparison of PSV at 45 degrees versus 60 degrees angles of insonation [35]. The radiologists consensus which is the commonest criteria used in various laboratories do not recommend a fixed angles however they recommend 60 degree or less [22]. In 2010, experts from the University of Washington Ultrasound Reading Center, published standardized ultrasound evaluation of carotid stenosis for clinical trials, these guidelines provided details on the technical consideration and emphasized on the use fixed angle of insonation at 60 degrees [24]. The ideal angle of insonation remains a topic of discussion [24, 35].

Furthermore, another important topic on the equipment that has received little attention is the probe frequency of the probe. The early research that established the velocity criteria which is still in use today utilized lower frequency transducers, as this was the most advanced technology available at the time. A good example is Dr Strandness who initiated the very first grading criteria in 1987 [8] in his study he used the probe with the frequency of 5MHZ, the criteria he formulated became foundational for subsequent researchers who developed additional grading systems [40]. In 2003 the consensus developed the criteria which are widely used in today’s time, and the normal value that were proposed during the Strandness time were adopted in 2003 and are still being used as reference even in the new era where the commonly used frequency is 7 MHz and above. This is likely the reason why other laboratories conducting studies today propose different cutoff points for the normal peak systolic velocity (PSV) of the internal carotid artery (ICA), such as 140 or 200 MHz [27, 41].

### Scanning protocols

In this review we identified a few institutions that have developed and published guidelines on scanning protocols and technical considerations. These include but are not limited to UT Southwestern, The American College of Radiology (ACR) and American Institute of Ultrasound in Medicine (AIUM), the Society for vascular ultrasound, and Columbia University in the project named Multi-Ethnic Study of Atherosclerosis (MESA).

- UT Southwestern came up with a comprehensive summary document entitled **"**Ultrasound—carotid doppler complete evaluation" the first version was developed in 2019, revised in 2022 and in 2024. The document provides an overview of the purpose, indications, and techniques for conducting carotid Doppler ultrasounds, along with specific criteria for evaluating carotid artery conditions [21].
- The ACR has established practice parameters for the performance of ultrasound examinations of the extracranial cerebrovascular system, detailing scanning techniques and indications for carotid ultrasound [42].
- Society for vascular ultrasound, which is society established in 1977, dedicated to advancing non-invasive vascular technology used in the diagnosis of vascular diseases provides comprehensive guidelines for conducting and reporting on extracranial cerebrovascular examinations, specifically using duplex ultrasound technology. The guidelines recommend the use of less than 60 degrees and whenever possible to keep it between 45 and 60 degrees. [30]
- American Institute of Ultrasound in Medicine (AIUM**):** The AIUM Practice Guidelines for the Performance of an Ultrasound Examination of the Extracranial Cerebrovascular, this guideline outlines the procedures for ultrasound imaging of the extracranial cerebrovascular system, including the common carotid, internal carotid, and vertebral arteries. It emphasizes the importance of using Doppler spectral analysis and color Doppler imaging to evaluate cerebrovascular abnormalities. The document also details indications for ultrasound examinations, such as evaluating patients with neurological symptoms or preoperative assessments for cardiovascular surgeries, the guidelines was first published in 2003, updated in 2012, 2016, 2018 and 2021. The focus is on ensuring high-quality imaging and adherence to standardized protocols, and interpretation criteria necessary for accurate diagnosis [42–46].

### Carotid intimal medial thickness (CIMT)

CIMT is extensively investigated as a surrogate marker for diagnosing subclinical atherosclerosis for risk assessment and tracking the progression of atherosclerosis for medical intervention [47]. It is believed that increased CIMT may be representative of generalized atherosclerosis in the body [48]. However, there is an inconsistent predictive value of CIMT which is likely attributed to the variability of CIMT measurement methodology like the image acquisition, and the CIMT mean calculation [49]. 

CIMT image acquisition differs between studies [49]. The acquisition of CIMT images varies in terms of the number of segments, sides, and angles used across different studies. Some research focused primarily on a single segment, particularly the distal common carotid artery (Figure), due to its accessibility [50, 51]. Other studies performed CIMT assessment at two segments including the common carotid artery and the internal carotid artery, others examined three segments: common carotid artery, carotid bulb, and internal carotid artery [52]. The discrepancy also exist in the number of imaging views to be taken, some studies examined the CIMT through a single anterior view while others utilized three angles from the anterior, lateral and posterior views [53, 54]. The quantification of CIMT measurements varies across studies. Some report the mean or maximum value from a single segment, while others provide the mean of the means or the mean of the maximum values from two or more segments [51].

Furthermore, some studies in literature used the far wall because the far wall measurement reflects the true wall thickness and is believed to be more accurate than the near-wall measures, however, Lisa Seekircher and his colleagues recommended the both near and far walls for measurement of the carotid intimal medial thicknesss [55]. Although CIMT is a common practice in vascular ultrasound laboratories for clinical and research purposes, there is no widely accepted ultrasound protocol for scanning and measurement of the CIMT. The existence of multiple approaches for measuring and scanning the CIMT creates confusion in the literature and also this leads to confusion among the end users. In this study we identified many institutions that attempted to develop carotid stenosis grading, however for the CIMT we identified multiple individual studies that tried to discuss the need for standardization of CIMT but only two consensus reports were identified and included in the seventeen reported studies [16, 31].

**Fig. 2:**
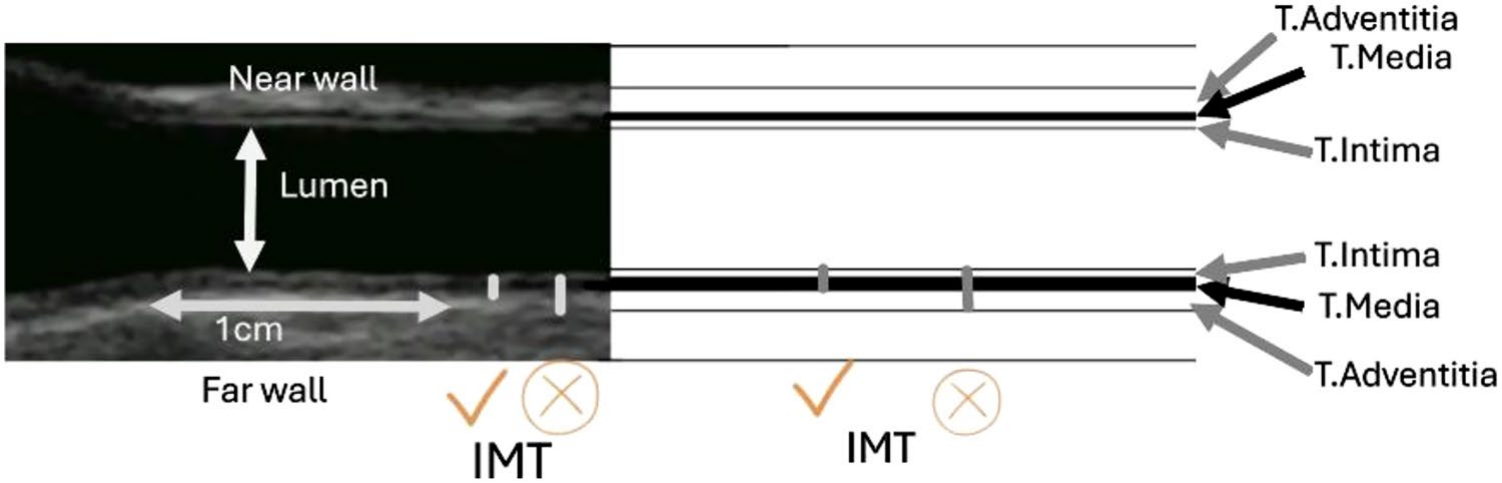
Common carotid artery (ultrasound and drawing) showing the layers of arterial walls and the lumen. (*A* Adventitia, *M* Media, *I* Intima, *NW* Near wall, *FW* Far wall, *L* Lumen, *IMT* Intimal medial thickness)

American Society of Echocardiography (ASE) Consensus Statement [16] and the Mannheim CIMT Consensus Report [31] were identified as group reports that were trying to address the issues of standardization and harmonization of CIMT scanning and measurements. According to the American Society of Echocardiography, the CIMT should be taken from three imaging angles in the longitudinal planes of anterior, lateral, and posterior. The Mannheim CIMT Consensus Report on the standardization of the CIMT recommends that the measurement should be performed on the far wall of the distal segment of CCA at least 5 mm away from its bifurcation. Both the Mannheim CIMT Consensus Report and the American Society of echocardiography suggested that CIMT more than 1.5 mm is considered as a plaque [51]. However, debate continues on the number of angles to be taken, which walls to be measured, and which and how many segments to be used for CIMT. This standard suggests that the IMT should be taken using at least 7MHZ and above [31]. Unified criteria are needed for harmonized practice in carotid atherosclerosis diagnosis [31]. Figure 2 shows the common carotid artery (ultrasound and drawing) displaying the layers of arterial walls as well as the lumen.

### Carotid plaques

Carotid plaque is a strong predictor of stroke [2], and is known as a focal protrusion encroaching into the arterial lumen by at least 50% of the surrounding IMT value, or with a thickness > 1.5 mm. [31] Studies have proved that the addition of carotid plaque description to the CIMT measurements improves the accuracy of carotid ultrasound in predicting cardiovascular events and stroke attacks [53, 56]. Carotid plaques indicate an advanced atherosclerosis process and are more prevalence at the proximal ICA and carotid bulb [57].

Carotid plaque can be measured as total plaque area, total plaque volume, or just by measuring the thickness [58, 59]. Plaques can also be characterized based on the appearance such as echogenicity, or smoothness of the surface. Plaque characterization by means of echogenicity, echotexture and echotexture provides a clue to differentiate “vulnerable” from “non-vulnerable” plaques and may help in risk stratification and planification of treatment intervention [60, 61]. Although current guidelines have defined degree of carotid stenosis as the key surrogate for stroke risk and indication of intervention, there is growing recognition of the importance of atherosclerotic plaque composition and morphology in determining stroke risk [62]. Defining cut-off values for carotid plaques as predictors of cardiovascular disease (CVD) presents various challenges, such as variability in measurement, the complexity of plaque features, plaque characteristics, morphology, lack of consensus on cut-off values and lack of standardization [63].

From this review, the majority of protocols identified in literature were focusing on the carotid stenosis grading criteria, however we identified two articles that have developed and validated the classification of plaques [17, 32]. In 2020, the American society of echocardiography released recommendations carotid plaque evaluation with ultrasound and they provided classification which classified the carotid plaques into categories from grade zero( normal) to grade three of a plaque more than 2.5 mm [17]. Four years later in 2024, Luca Saba et al. published a novel plaque grading named Carotid Plaque RADS, which provide additional details on the morphological assessment of the plaque. Plaque RADS classification ranges from grade 1 which indicates normal vessel to grade 4 which indicate a complicated plaque (more vulnerable plaque). This grading mechanism was not only developed for ultrasound, it is also applicable to other imaging modalities, like computed tomography and magnetic resonance imaging [32]. In 1998 G Geroulakos classified the plaques into five types: Type 1 for uniformly echolucent, type 2 for predominantly echolucent, type 3 for predominantly echogenic, type 4 for uniformly echogenic and type 5 for plaques that are heavy calcified with acoustic shadows. Type 1 plaque which is uniformly echolucent was found in 90 percent of symptomatic patients [64].

Recent advancements in ultrasound technology have significantly enhanced the assessment of carotid plaques, particularly in diagnosing atherosclerosis, however, there is a lack of consensus in reporting and interpreting carotid plaque features.

### Carotid stenosis grading

Stroke is the third largest cause of death and the primary cause of long-term disability. Severe stenosis of the carotid artery is a significant cause of stroke [65]. Stroke is a severe public health burden that is expected to worsen in the coming decades as population demographics shift, particularly in emerging countries [66].

In this review, 17 articles that were identified described carotid ultrasound. However, 14 of them focused on carotid stenosis grading while three of them focused on the carotid plaques and intimal medial thickness. The Society of Radiologists in Ultrasound Consensus Conference identifies the primary parameters for assessing carotid stenosis as the Internal Carotid Artery Peak Systolic Velocity (ICA PSV), the Internal Carotid Artery End Diastolic Velocity (ICA EDV), and the PSV ratio of the ICA to the common carotid artery. Additionally, the Joint Recommendations from the United Kingdom suggest using the peak systolic ICA to end diastolic Common Carotid Artery ratio (known as the St Mary’s ratio) as a more reliable index for grading stenosis in deciles [67]. Multiple studies suggested the use of additional criteria such as morphological description of the plaque and post stenotic flow behaviors have been proposed for grading carotid stenosis to overcome diagnostic errors that have been reported in different clinical population based and hospital-based studies. [68, 69] Currently there is no globally standardized carotid stenosis criteria. The lack of internationally accepted ultrasound criteria for describing the degree of stenosis has pushed different professional societies in developed countries to come up with their own local criteria and some have undergone internal validation.

Duplex ultrasound serves as the primary noninvasive diagnostic modality for the detection, assessment, and monitoring of carotid artery stenosis, attributed to its affordability, high resolution, and extensive accessibility. However, as highlighted in this review, there exists considerable variability in the application of ultrasound diagnostic criteria for carotid artery stenosis across different counties and departments. Currently, no internationally recognized standard exists for the classification of carotid stenosis. Variations in ultrasound criteria may lead to significant misclassifications of disease severity, potentially resulting in inappropriate referrals which could have serious adverse consequences [33]. Some researchers suggest that morphological assessment using B-mode images and color flow imaging should be the main criteria for low and moderate degrees of stenosis [68, 70].

Since 1980s, the carotid duplex criteria established by the University of Washington have been extensively utilized and adapted in vascular laboratories globally [71]. The Strandness criteria that was developed in 1987 by Dr. Strandness and his team from the University of Washington. (Table 3) [8]. These criteria were mainly based on peak systolic and end-diastolic velocity, as well as spectral broadening. Carotid stenosis was classified into 6 categories: normal, 1% to 15%, 16% to 49%, 50% to 79%, 80% to 99%, and occluded. Following the original publication, numerous organizations have developed consensus criteria to enhance the classification of stenosis severity; numerous carotid diagnostic ultrasound criteria methods for classifying the severity of stenosis have been suggested. [23, 72]

**Table 3:** University of Washington criteria for carotid stenosis with Duplex ultrasound [8]

Stenosis (%)	PSV (cm/s)	EDV (cm/s)	Flow characteristics
1 to 15	< 125	< 140	No spectral broadening
16 to 49	< 125	< 140	Minimal spectral broadening
50–79	> 125	< 140	Marked spectral broadening
80–99	> 125	> 140	Marked spectral broadening
Occlusion	N/A	N/A	No internal carotid flow signal

### The German Society of Ultrasound in Medicine criteria (DEGUM)

In 1986, The German Society of Ultrasound in Medicine (known by its acronym DEGUM) developed what is known as DEGUM criteria which were revised in 2010. The DEGUM criteria were initially aligned with the definitions used in the European Carotid Surgery Trial (ECST) for grading carotid stenosis based on local diameter reduction percentage [6]. However, to address discrepancies with the North American Symptomatic Carotid Endarterectomy Trial (NASCET) methodology, which uses distal diameter reduction percentage, a revision was initiated. This led to the adoption of NASCET standards as the benchmark for grading carotid stenosis in Germany. The first major revision occurred in 2010, where the DEGUM ultrasound criteria were updated to incorporate a multi-parametric approach, combining both Doppler and imaging criteria to enhance diagnostic accuracy [14]. While the DEGUM criteria aim to provide a comprehensive framework for grading carotid artery disease through a multiparametric approach, they face substantial criticism regarding their diagnostic sensitivity, complexity, and variability [73].

### The Society of Radiologists in Ultrasound (SRU) criteria

Following the findings of multiple clinical trials, the Radiologist Society issued a consensus statement on carotid stenosis grading with duplex ultrasonography in 2003 [22]. (Table 4) The criteria established by the Society of Radiologists in Ultrasound (SRU), are the most frequently utilized standards [33]. However, these criteria have faced criticism for their mainly reliance on peak systolic velocities, a factor that can lead to over or under estimation of carotid stenosis when considered alone [74]. The Society of Radiologists in Ultrasound (SRU) criteria aimed to standardize the diagnosis of carotid artery stenosis, enhance accuracy in clinical practice, and ultimately improve patient outcomes related to stroke prevention. According to the SRU criteria stenosis levels are categorized as normal, < 50%, 50–69%, ≥ 70% to near occlusion, near occlusion, and total occlusion based on gray-scale and Doppler findings [22]. Key diagnostic indicators include peak systolic velocity (PSV) and plaque presence, with additional parameters like the ICA-to-common carotid artery PSV ratio and end-diastolic velocity used when necessary. Specific PSV thresholds define stenosis severity: normal (< 125 cm/sec with no plaque), < 50% stenosis (< 125 cm/sec with plaque), 50%-69% stenosis (125–230 cm/sec with plaque), ≥ 70% stenosis (> 230 cm/sec with plaque), near occlusion (markedly narrowed lumen), and total occlusion (no detectable lumen or flow) [22, 33]. Although these criteria has been the most commonly used criteria, substantial criticisms remain regarding its accuracy, standardization, and lack of detailed criteria for plaques grading or plaques characterization [33].

**Table 4:** DUS Criteria for internal carotid stenosis based on the Radiologist consensus panel (2003) [22]

Degree of stenosis	Primary parameters		Additional parameters	
	ICA PSV (cm/sec)	Plaques estimation (%)	ICA/CCA PSV ratio	ICA EDV (cm/sec)
Normal	< 125	None	< 2.0	< 4.0
< 50%	< 125	< 50	< 2.0	< 40
50–69%	125–230	≥ 50	2.0–4.0	40–100
≥ 70% but less than near occlusion	> 230	≥ 50	> 4	> 100
Near occlusion	High, low or undetectable	Visible	Variable	Variable
Total occlusion	Undetectable	Visible, no detectable lumen	Not applicable	Not applicable

In 2005 Dr. G. M. Hathout and colleagues introduced the Sonographic NASCET Index (SNI). The aimed was to create a new Doppler parameter that would provide greater correlation with angiographic findings in assessing carotid artery stenosis, particularly in alignment with the methodologies established by the North American Symptomatic Carotid Endarterectomy Trial (NASCET) [75].

The development of the SNI involved deriving a Doppler parameter that incorporates flow velocity measurements from both the proximal internal carotid artery (ICA) at the site of maximal stenosis and the distal ICA, applying the mass balance principle to enhance diagnostic accuracy compared to traditional methods like peak systolic velocity (PSV) alone. This innovative approach aimed to improve sensitivity and specificity for detecting significant carotid stenosis (≥ 70%) and was validated through retrospective analyses against conventional angiographic standards. Few years later the SNI underwent revisions to address limitations and improve its predictive capabilities, leading to the introduction of the revised Sonographic NASCET Index (rSNI) in 2014. This revision was also developed by Dr. Hathout and his team, further refining the method for better clinical applicability [76]. While the Sonographic NASCET Index has not been specifically endorsed by any professional society, it is designed to complement and enhance existing methodologies recognized by organizations like the SRU. Its development reflects an effort to improve diagnostic accuracy in carotid artery assessment consistent with established trials such as NASCET.

### Joint recommendations for reporting carotid ultrasound investigations in the United Kingdom

In 2009, a similar initiative was established in the United Kingdom, which involved forming a dedicated working group aimed at standardizing the acquisition, interpretation, and reporting of carotid ultrasound examinations to ensure consistency across practices [23].

The Joint Recommendations for Reporting Carotid Ultrasound Investigations in the UK, published in 2009, aimed to standardize the grading of carotid artery stenosis using ultrasound. This initiative was driven by the need to address discrepancies in ultrasound criteria across different centers, which arose from variations in grading methodologies used in major clinical trials like NASCET and ECST. The recommendations were developed by a joint working group comprising the Vascular Society of Great Britain and Ireland and the Society for Vascular Technology of Great Britain and Ireland. The guidelines emphasized using peak systolic velocity (PSV) and end-diastolic velocity (EDV) to classify stenosis. The use of the St Mary's Ratio was recommended for grading stenosis in deciles beyond 50% (Table 5). The recommendations sought to create a unified approach to reporting, thereby reducing variability among vascular units. They promoted consistent measurement techniques, such as maintaining a Doppler angle between 45 and 60 degrees during assessments [23]. A recent audit conducted in 2024 assessed adherence to these recommendations across various vascular centers in the UK and Ireland. The findings indicated that while a majority (70%) of centers reported using the 2009 guidelines, there remained significant variability in practices not covered by these recommendations such as the use of EDV which were not explicitly detailed in the original guidelines [77].

**Table 5:** Joint Recommendations for diagnostic criteria for Reporting Carotid Ultrasound Investigations in the United Kingdom [23]

Percentage stenosis (NASCET)	Internal carotid PSV (cm/s)	PSV ratio ICA/CCA	St Mary's ratio ICA/CCA edv)
< 50	< 125	< 2	< 8
50–59	> 125	2–4	8 to 10
60–79	> 230	> 4	11 to 13
70–79	> 230	> 4	14 to 21
80–89	> 230	> 4	22 to 29
> 90 but less than near occlusion	> 400	> 5	> 30
Near occlusion	High, low-string flow	Variable	Variable
Occlusion	No flow	Not applicable	Not applicable

In 2010, a team of researcher from the Eugene Strandness Vascular Laboratory, Department of Surgery, University of Washington published a paper entitled "Standardized Ultrasound Evaluation of Carotid Stenosis for Clinical Trials: University of Washington Ultrasound Reading Center" the paper highlighted the significance of standardized protocols for evaluating carotid artery stenosis (CS) through ultrasound, especially in clinical trial settings [24]. It emphasizes duplex Doppler ultrasonography as a reliable method for assessing carotid stenosis and offers a comprehensive approach to carotid ultrasound with a particular focus on clinical trials [24]. The study found that variations in Doppler angles can lead to discrepancies in peak systolic velocity (PSV) measurements and therefore used a standard value of 60 degrees. However, other researchers propose the use of variable angles less than 60 degrees. Whal Lee in his paper proposed that the angle of insonation should be between 30 and 60 degrees [36]. A study done by Kari A Campbell revealed that there a statistically significant difference in the PSV measurements taken while taken at varying Doppler angles and suggested the use of the lower doppler angle as possible because the more the angle increases the higher the PSV [78].

The study from the University of Washington Ultrasound Reading Center stresses that standardization of the Doppler angle and the carotid ultrasound protocols in general can mitigate the differences in practice and improve diagnostic reliability and enhance the consistency of ultrasound evaluations across different clinical settings, ultimately improving patient outcomes by ensuring accurate diagnosis and treatment planning. [24]

### Standards of the Polish Ultrasound Society – update. Examination of extracranial carotid and vertebral arteries

The standards were first established in 2011, focusing on various applications of ultrasonography across medical specialties. The guidelines aimed to standardize ultrasound practices in Poland to enhance diagnostic accuracy and patient safety. The protocols were updated in 2014, incorporating new findings from literature and clinical experiences. This update specifically addressed the examination of extracranial carotid and vertebral arteries, emphasizing the importance of Doppler ultrasound in assessing stroke risk due to carotid artery stenosis **(**Table 6**)**. The standards detail specific protocols for conducting ultrasound examinations of the carotid and vertebral arteries. These revised criteria propose maintaining an angle of insonation at 60° during examinations to reduce measurement errors, a practice that remains contentious across various literature standards. Additionally, the standards highlight the need to characterize atherosclerotic plaques, especially identifying hypoechoic plaques, which suggest a higher risk of stroke. Moreover, they emphasize hemodynamic assessment over morphological measurements, underlining the significance of blood flow dynamics in evaluating stenosis severity. The updated Standards of the Polish Ultrasound Society is one of the comprehensive guidelines, taking into account criticisms raised by researchers in recent literature [19, 67]. According to these standards, the intimal medial thickness of 0.9 mm in females and greater in males is deemed pathological. While the updated standards from the Polish Ultrasound Society offer significant details in terms of the standardization of examination protocols, challenges such as variability in measurements, persists. The patient categories based on the size of stenosis which ranged from category one to three which appear in these standards has not been reported in other standards. This shows the lower updake of this practice [19].

**Table 6:** Standards of the Polish Ultrasound Society – update. Examination of extracranial carotid and vertebral arteries [19]

Vessel Segment	PSV	EDV
CCA	0.8–1.2 m/s	0.1–0.3 m/s
ICA	0.8–1.2 ms	Do 0.3 m/s
ECA	0.8–1.2 m/s	Do 0.25 m/s
VA	< 0.6 m/s	0.005–0.02 m/s

*CCA* Common carotid artery, *ICA* Internal carotid artery, *ECA* External carotid artery, *VA* Vertebral artery

### The Swedish criteria for grading carotid stenosis

The Swedish criteria (Table 7) for grading carotid stenosis differ significantly from American criteria,

**Table 7:** Standard criteria for grading carotid stenosis in Sweden [35]

Systolic maximal velocity		Degree of stenosis	
Angle < 45	Angle 55–60	ECST (%)	NASCET (%)
< 1.1 m/s	< 1.3 m/s	< 50	< 20
1.1–1.6 m/s	1.3–2.2 m/s	50–69	20–49
1.7–2.0 m/s	2.3–3.1	70–79	50–69
≥ 2.1 m/s	≥ 3.2 m/s	80–99	70–99
No signal	No signal	Occlusion	Occlusion

particularly in the methodologies and specific thresholds used for duplex ultrasound measurements. The Swedish guidelines emphasize the importance of angle-dependent measurements and the assessment of plaque burden alongside flow velocities [35]. The Swedish guidelines differ from those from the Society of Radiologists in Ultrasound (SRU) and recommend different PSV cutoffs for grading stenosis based on the angle of insonation used. They emphasize that the 45 and 60 degrees yield different measurements of PSVs. The ultrasonography criteria used in Sweden (and Germany) differ significantly from those recommended in North America and the United Kingdom for the same level of angiographic stenosis [35]. The discrepancies underscore the significance of local consensus.

#### IAC vascular testing communication: updated recommendations for carotid stenosis interpretation criteria

These guidelines focus on standardizing criteria across accredited testing facilities and utilizing rigorously validated metrics to assess the severity of internal carotid artery (ICA) stenosis, particularly emphasizing velocity thresholds for identifying greater than 50% stenosis more accurately. The updated criteria suggest raising the peak-systolic velocity (PSV) threshold for diagnosing ≥ 50% ICA stenosis from 125 cm/s to 180 cm/s. This adjustment aims to enhance sensitivity and specificity when compared to traditional methods, which have shown significant overestimation in stenosis severity [10].

### Strengths and limitations

One of the strengths of this review is the comprehensive nature of the included studies, and the fact that we selected only those that have been developed at the institutional level or professional society level which increase the reliability of the protocols included in this study. However, limitations include the heterogeneity of study designs and elements included in their protocols, which complicates direct comparisons. Many studies lacked concentrated on one aspect of carotid ultrasound, those that focused on carotid stenosis, had tendance to ignore the details about intimal medical thickness protocols and plaques characterization, and vice versa. This shows the need for a holistic approach in defining protocols for carotid ultrasound for harmonized scanning and reporting style.

### Implications for future research

The findings highlight several gaps in the literature, particularly concerning the harmonization of carotid ultrasound practice. Future research should focus on standardized protocols for carotid ultrasound scanning as well as reporting, not only focusing on the carotid stenosis but also covering other concepts such as carotid plaques characterization, hemodynamics factors, intimal medial thickness measurement to facilitate uniformity in disease management, early detection, and classification of stenosis and plaques across the world and within the individual countries and departments. Additionally, establishing a multidisciplinary consensus that involve medical imaging personnel and clinicians managing patients with and at risk of stroke and cardiovascular disease, stake holders and policy makers is important for the attainment standardized practice and effectiveness of the test across diverse populations.

### Conclusion

Prevention is the ideal treatment for cerebrovascular disease. Park emphasized on the importance of early diagnosis and screening of high-risk patients with carotid stenosis and carotid plaques. Atherosclerosis is silent in nature, and 80% of stroke cases occur in asymptomatic individuals, this mark the importance of screening programs targeting high-risk patients [79].

Duplex ultrasound is a powerful tool for evaluating carotid vessels. Carotid ultrasound is useful not only for identifying carotid artery stenosis, carotid ultrasound may be used for risk assessment for ischemic stroke beyond measurement of luminal stenosis, it can also be used for detecting atheromatous plaque as well as the intimal medial thickness changes. With advancement in technology, patients risk stratification can be made by identification of plaque morphology, plaque size and plaque ulceration. Several studies have reported that some patients with high-grade stenosis may be asymptomatic and conversely, there exists a substantial proportion of patients with what is known as non-significant stenosis (< 50%) who develop stroke symptoms. Thus, imaging technologies that better capture morphologic details of plaque morphology should be considered. Although a lot have been written on the use of carotid Duplex ultrasound in the diagnosis of atherosclerosis, there is no consensus about the universal comprehensive protocols on scanning and reporting carotid ultrasound that covers all the steps of carotid ultrasound including the carotid intimal medial thickness, carotid plaques and carotid stenosis in a single guideline which may guide the end users and avoid discrepancies in the carotid ultrasound practices.

MRI and CT scan are other imaging modalities that are commonly used in carotid stenosis grading and plaque characterization and may be more sensitive than ultrasound in the detection and quantification of carotid atherosclerosis, however, these modalities are still very few in developing countries and are inaccessible at sub-national hospitals. Ultrasound is a non-invasive modality, widely available, accessible and affordable even in low-income countries. The challenges that hinder the utilization of ultrasound for early detection of atherosclerosis for stroke and cardiovascular diseases prevention programs are related to the health workforce trainings and lack of standardized scanning protocols and reporting protocols. As cardiovascular disease and stroke remain the leading causes of death globally, prevention and early detection of atherosclerosis is crucial, to inform planning of interventions. Currently, the ultrasonography criteria used in Sweden and Germany differ significantly from those advised in North America and the United Kingdom [35]. In Africa, there are proven regional guidelines. There is a need for standardized approach toward early detection of atherosclerosis which have been repeatedly reported as silent killer [80–82]. The duplex carotid ultrasound remains the most preferred and useful initial test in detecting the degree of subclinical carotid atherosclerosis, and has the advantage of being noninvasive, safe and use non-ionizing radiations [3, 71].There is a need for a multiparametric approach that would incorporate all diagnostic parameters to improve accuracy in early detection of atherosclerosis. The professional bodies advocate for standards that enhance diagnostic accuracy.

In conclusion, while carotid ultrasound is a valuable tool for assessing carotid artery stenosis due to its non-invasive nature and convenience, the discrepancies between different scanning techniques, measurements and cut-off values agreement highlight the necessity for standardized diagnostic criteria to ensure consistent patient care and accurate assessments across different clinical settings. Therefore, while there are no existing universal criteria for scanning and reporting the carotid ultrasound, there is a need for at least national protocols to ensure the uniformity of practice for harmonized patients care, timely referral and timely intervention to reduce the impact of atherosclerotic diseases in the country. Carotid atherosclerosis should be detected and treated early, and more studies are needed to determine the best practice approaches for Duplex carotid ultrasound screening in high-risk patients such as hypertensive and diabetic patients. It is important to have standardized interdisciplinary and intercontinental protocols to guide practitioners from low-income towards consistent and standardized practice. WHO and its partners should play a central role in coordinating imaging professional societies from different regions and come up with universal accepted criteria for carotid duplex ultrasound. This will lead to the reduction of stroke and cardiovascular incidence which are the leading causes of death worldwide and more prevalent in low-income countries.

## Data Availability

Not applicable.

## Declarations

### Artwork and illustrations guidelines

Figure 2 consists of an ultrasound image sourced from the First Author’s clinical archive, alongside illustrations created using OneNote free software.
